# Genetic Testing in Patients with Neurodevelopmental Disorders: Experience of 511 Patients at Cincinnati Children's Hospital Medical Center

**DOI:** 10.1007/s10803-021-05337-6

**Published:** 2021-11-13

**Authors:** Xiaoli Du, Jennifer Elaine Glass, Stephanie Balow, Lisa M. Dyer, Pamela A. Rathbun, Qiaoning Guan, Jie Liu, Yaning Wu, D. Brian Dawson, Lauren Walters-Sen, Teresa A. Smolarek, Wenying Zhang

**Affiliations:** 1 Division of Human Genetics, Cincinnati Children’s Hospital Medical Center, 3333 Burnet Avenue, Cincinnati, OH 45229 USA; 2 Department of Pediatrics, University of Cincinnati College of Medicine, Cincinnati, OH USA; 3 Present Address: Pathology and Laboratory Medicine, Ann and Robert H. Lurie Children’s Hospital of Chicago, Chicago, IL USA

**Keywords:** Autism spectrum disorder (ASD), Copy number variant (CNV), Fragile X, *MECP2*, Neurodevelopmental disorders

## Abstract

Our institution developed and continuously improved a Neurodevelopmental Reflex (NDR) algorithm to help physicians with genetic test ordering for neurodevelopmental disorders (NDDs). To assess its performance, we performed a retrospective study of 511 patients tested through NDR from 2018 to 2019. SNP Microarray identified pathogenic/likely pathogenic copy number variations in 27/511 cases (5.28%). Among the 484 patients tested for Fragile X *FMR1* CGG repeats, a diagnosis (0.20%) was established for one male mosaic for a full mutation, a premutation, and a one-CGG allele. Within the 101 normocephalic female patients tested for *MECP2*, two patients were found to carry pathogenic variants (1.98%). This retrospective study suggested the NDR algorithm effectively established diagnoses for patients with NDDs with a yield of 5.87%.

**Supplementary Information:**

The online version contains supplementary material available at 10.1007/s10803-021-05337-6.

Neurodevelopmental disorders (NDDs) are a group of disorders with impairments of the growth and development of the central nervous system, including autism spectrum disorders (ASD), intellectual disability (ID), developmental delay (DD), etc. NDDs can present as neuropsychiatric problems, impaired motor function, learning difficulties, language delay or non-verbal communication and affect > 3% of children worldwide (Mithyantha et al., 2017; South et al., 2013; Waggoner et al., 2018). Determining the genetic etiology of a child’s NDD can ensure early interventions, access to appropriate services, prognostic information, and accurate recurrent risk assessment. Genetic testing has been recommended by several professional societies, such as the American College of Medical Genetics and Genomics (ACMG), the American Academy of Neurology (AAN) and Child Neurology Society (CNS), and the American Academy of Pediatrics (AAP), for patients with NDDs (Filipek et al., 2000; Hyman et al., 2020; Manning et al., 2010; Michelson et al., 2011; Miller et al., 2010; Moeschler et al., 2014; Schaefer et al., 2013; South et al., 2013). However, despite recommendations for genetic testing in children with ASD/ID/DD, a survey study of 3371 families with children suffering from ASD/ID/DD in the United States showed the majority (68%) of these children did not undergo recommended genetic testing (Kiely et al., 2016).

Genetic testing for NDDs can be difficult for clinicians to navigate, especially for non-geneticists, and expensive for families and the health care system to afford. Chromosome microarray (CMA) for copy number changes has been recommended as the first-tier clinical diagnostic test for children with NDDs (Manning et al., 2010; Miller et al., 2010; South et al., 2013; Waggoner et al., 2018). Fragile X syndrome is one of the most common causes of X-linked intellectual disability and is caused by an expansion of a trinucleotide repeat region in the promoter of the *FMR1* gene (Mannermaa et al., 1996; Mila et al., 2018). Rett syndrome is caused by mutations in the *MECP2* gene and is a known genetic cause of X-linked autism and ID in normocephalic females (Vidal et al., 2019). Pathogenic variants in *PTEN* have been reported with a prevalence of 8.3% and 12.2% in the ASD-macrocephaly and DD/ID-macrocephaly populations (Varga et al., 2009).

Historically, most patients with NDDs at our institution had concurrent orders for Fragile X testing, chromosome analysis, and SNP microarray. *MECP2* test was only ordered for female children with typical Rett syndrome cognitive and neurologic phenotypes. The patient with atypical Rett syndrome might not be diagnosed by molecular technology (Schaefer et al., 2013). We noticed few orders for *PTEN* sequencing in patients with NDDs and macrocephaly, likely due to unawareness of the availability of *PTEN* testing and the complexities of ordering a send-out test.

Therefore, to streamline test ordering and decrease the cost of genetic testing in patients with NDDs, we developed a more complete, user-friendly, and cost-efficient reflexive testing algorithm, named the Neurodevelopmental Reflex (NDR) genetic test. The original NDR algorithm was separated for patients with or without macrocephaly. Those without macrocephaly started with CMA, and proceeded to Fragile X analysis if CMA was not positive (normal, likely benign, or variant of unknown clinical significance). Those with macrocephaly, as stated by the ordering clinician, started with *PTEN* sequencing. If *PTEN* sequencing was normal or found only a variant of unknown clinical significance, testing proceeded to Fragile X analysis. If Fragile X testing was negative for a full mutation (normal, grey zone, or premutation) then testing proceeded to CMA. After utilizing this algorithm for a year, we analyzed our outcomes and determined that *PTEN* sequencing was having a far lower than expected positivity rate in this population, and the algorithm was reformulated to improve its efficiency.

The improved NDR (Fig. 1), which we are evaluating in this study, puts CMA as the first line test for all, with Fragile X as the 2nd line for all (if CMA is not positive). If Fragile X analysis was negative for a full mutation (normal, grey zone or premutation repeats), patients noted to be macrocephalic would receive *PTEN* sequencing and normocephalic female patients would receive *MECP2* sequencing.

**Fig. 1:**
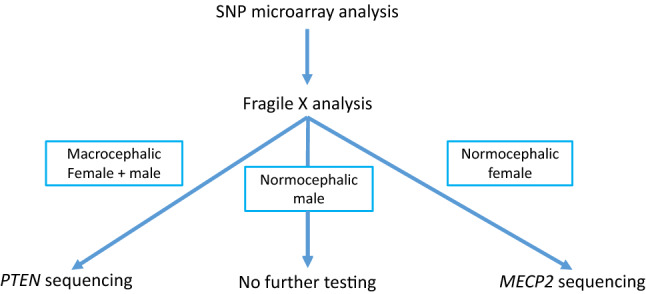
The algorithm of Neurodevelopmental Reflex (NDR) genetic testing. In this NDR algorithm, the SNP microarray analysis is the first-tier testing, followed by Fragile X analysis, and the *PTEN* or *MECP2* gene sequencing. *PTEN* gene sequencing is applied to macrocephalic patients (as reported by the ordering clinician) with both non-positive SNP microarray and Fragile X testing results. *MECP2* gene sequencing is applied only to normocephalic female patients with both non-positive SNP microarray and Fragile X testing results

In the current analysis, we hope to share our experience in the genetic diagnosis of patients with NDDs while we examine and summarize the clinical utility of this reflexive testing algorithm, which aligns with testing recommended by the ACMG for the genetic diagnosis of patients with NDDs (Moeschler et al., 2014; Schaefer et al., 2013; South et al., 2013). We hope to provide a model of how to streamline a genomic testing algorithm that meets ACMG recommendations in which pediatricians can order a single test for children with NDDs.

## Methods

### Patient’s Enrollment

A retrospective study of 511 individuals who had a NDR reflex panel ordered through Cincinnati Children's Hospital Medical Center (CCHMC) from January 2018 to April 2019 was conducted to evaluate the performance of the test and positivity rate. This study was approved by CCHMC Institutional Review Board. Subjects' data were obtained in a de-identified state. In this investigation, the patient’s ages ranged from 3 months to 35 years old and the sex ratio of male/female was 2.8:1. Testing was ordered by clinicians from a broad range of specialties and test indications were extracted from the patients’ medical record.

### DNA Extraction and SNP Microarray Analysis

Genomic DNA was extracted either automatically using the Chemagic™ Magnetic Separation Module I instrument (PerkinElmer, Waltham, MA) or manually using Qiagen Puregene kits (Qiagen, Germany). Microarray analysis was performed using the Infinium Assay with the Illumina CytoSNP-850Kv1.2 BeadChip platform (Illumina, San Diego, CA).

### Fragile X

Isolated genomic DNA was assayed for CGG-repeat expansion of the *FMR1* locus by analysis of DNA fragments generated by the AmplideX FMR1 polymerase chain reaction (PCR) and separated by capillary electrophoresis ABI 3500XL Genetic Analyzer (Applied Biosystems, Foster city, CA). Normal and mutation categories of *FMR1* alleles were determined according to the ACMG guidelines with the normal repeat size as 5–44, gray zone as 45–54, premutation as 55–200, and full mutation > 200. Full mutations were confirmed by Southern blot.

### PTEN and MECP2 PCR and Sequencing

The entire coding region and exon/intron boundaries of genes were analyzed by PCR and bidirectional sequencing for *PTEN* (NM_000314.4) and *MECP2* (NM_004992.2) according to manufactory instructions (Roche, Indianapolis, IN).

### Blood Chromosomes

Chromosomal analysis was performed according to standard procedures using GTG-banding.

Detailed methodologies are listed in the supplementary data and available on request.

## Results

**Fig. 2:**
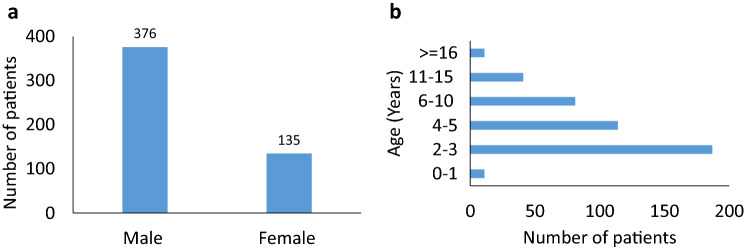
Description of the 511 patient’s cohort from January 2018 to April 2019. **a** A total of 511 patients tested at our institution from January 2018 to April 2019 included 376 males and 135 females. **b** Age ranged from a few months to 35 years old. The majority of the patients were under 5 years old

The NDR algorithm sets SNP Microarray as the first-tier test and reflexes to Fragile X testing if the result is not positive, then reflexes to *MECP2* sequencing for normocephalic female patients and *PTEN* sequencing for patients with macrocephaly (a head circumference > 98%ile) if the Fragile X result is not full mutation (Fig. 1). Common clinical indications for patients in our cohort included autism spectrum disorders, developmental delay, speech delay, intellectual disability, gross motor delay, mixed receptive-expressive language disorder, epilepsy, and/or dysmorphic features. We analyzed variants detected by this NDR algorithm on patients with neurodevelopmental disorders with samples received from January 2018 to April 2019 at CCHMC. A total of 511 patients (age 3 months – 35 years old, median 3 years) were tested. 376 (73.6%) were male and 135 (26.4%) were female. More than 36% patients (188 out of 511) were in the age group of 2 to 3 years. 22.3% patients (114 out of 511) were in the age group of 4 to 5 years. 15.8% patients (81 out of 511) were 6 to 10 years old. 14.8% patients (76 out of 511) were below 2 years (Fig. 2).

**Fig. 3:**
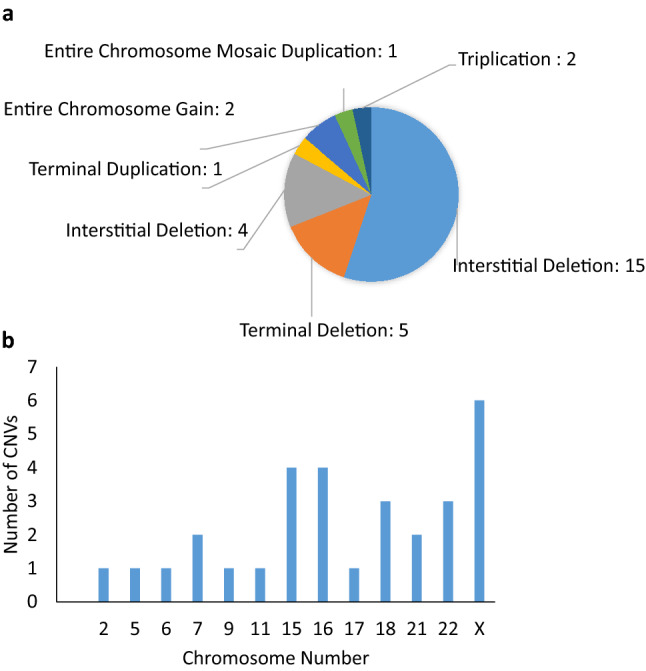
Clinically significant CNVs identified by SNP microarray analysis in 511 patients. **a** Classifications and frequency of 30 reportable CNVs in 27 patients through SNP microarray analysis. The majority of CNVs are interstitial deletions (15/30), followed by terminal deletions (5/30), interstitial duplications (4/30), entire chromosome gain and mosaic duplications (3/30). **b** Accumulated numbers of CNVs on different chromosomes. The most often affected chromosomes are X, 15 or 16

2826 CNV variants were identified through SNP Microarray analysis in the 511 patients. 30 CNVs were pathogenic/likely pathogenic and explained the patient’s clinical phenotype (1.06%). 4.5% were CNVs of uncertain clinical significance (127). SNP Microarray results provided a diagnosis with clinical significance or likely clinical significance in 27 out of 511 cases (5.28%), and uncertain clinical significance in 53 out of 511 cases (10.37%). The majority of the 30 CNVs from 27 patients reported were chromosome interstitial deletions (15/30), followed by terminal deletions (5/30), interstitial duplications (4/30), entire chromosome gain (2/30), and triplication (2/30) (Fig. 3a). The most frequent CNVs identified in this study were from chromosome X, 15 or 16 (Fig. 3b). The most commonly seen syndrome diagnosed by CMA in this study is 15q13.3 microdeletion syndrome from four patients (Pts 283, 361, 506, 453, Table 1).

**Table 1:** Summary of clinically significant copy number variants (CNVs) identified in 27 out of 511 patients with NDDs

Patient ID	Clinical information	ISCN nomenclature	Chromosome locations/Size of alterations	Candidate genes	Genetic diagnosis	Studies that previously report CNVs
89	2 year old male with global developmental delay, hypotonia	arr[GRCh37] **2q23.3q24.1(152826896_157180476) × 1**	2q23.3–2q24.1: 4.4 Mb deletion	*CACNB4, KCNJ3, PRPF40A, FMNL2*	2q23.3–2q24.1 deletion	This study
319	4 year old male with global developmental delay, speech delay, dysmorphic craniofacial features	arrh[GRCh37] **5p15.33(25328_1721527) × 3,** **18p11.32(13034_2243990) × 1**	5p15.33: 1.7 Mb duplication; 18p11.32: 2.2 Mb deletion	*SLC6A19, TERT*	Unbalanced translocation der(18)t(5;18)(p15.33;p11.32)	This study
267	10 year old male with mild intellectual disability, hypotonia, abnormal MRI (small left medial cerebellar hemisphere, hypoplastic right olfactory bulb and incomplete myelination of temporal lobes), gross motor impairment, fine motor impairment	arr[GRCh37] **6q16.1q16.3(95913318_104980478) × 1**	6q16.1–6q16.3: 9.1 Mb deletion	*SIM1, GRIK2, FBXL4, NDUFAF4*	6q16.1–6q16.3 deletion	This study; CNVs overlapping with this deletion reported previously (Bonaglia et al., 2008; Kasher et al., 2016; Strunk et al., 2016)
29	3 year old male with congenital atresia of esophagus, tracheomalacia, tracheoesophageal fistula, feeding problem, gastroesophageal reflux disease, abnormal posture, oropharyngeal phase dysphagia, mixed receptive-expressive language disorder	arr[GRCh37] **7q11.22(69564501_69795311) × 1**	7q11.22: 230.8 Kb deletion	*AUTS2*	AUTS2 syndrome	(Amarillo et al., 2014; Beunders et al., 2016; Beunders et al., 2013; Liu et al., 2015)
303	3 year old male with global developmental delay, aortic arch hypoplasia, moderate right, mild left branch pulmonary artery stenosis	arr[GRCh37] **7q11.23(72722981_74141840) × 1**	7q11.23: 1.4 Mb deletion	*ELN*	Williams-Beuren syndrome	(Pober, 2010; Samanta, 2017)
367	6 year old male with transient neonatal hypoglycemia, muscle weakness, oral phase dysphagia, speech disturbance, motor skills developmental delay, feeding difficulties, astigmatism, lack of coordination, hyperopia, sensory processing difficulty	arr[GRCh37] **9p24.3p23(46587_13422337) × 1**	9p24.3–9p23: 13.4 Mb deletion	Many (genes)	9p deletion syndrome	(Bayat et al., 2018; Sivasankaran et al., 2016; Spazzapan et al., 2016; Tassano et al., 2016)
270	18 year old male with autism	arr[GRCh37] **11p11.2(44222462_44236652) × 1**	11p11.2: 14 Kb deletion	*EXT2*	Hereditary multiple osteochondromas	(D’Arienzo et al., 2019; Jennes et al., 2008)
283	21 month old female with developmental delay, decreased motor activity, gross motor development delay	arr[GRCh37] **15q13.2q13.3(30657952_32833659) × 1**	15q13.2–15q13.3: 2.2 Mb deletion	*CHRNA7*	15q13.3 microdeletion syndrome	(Ben-Shachar et al., 2009; Hoppman-Chaney et al., 2013; Lowther et al., 2015; Ziats et al., 2016)
361	5 year old male with closed fracture of shaft of clavicle, mixed receptive-expressive language disorder, speech disturbances, autism spectrum disorder, global developmental delay	arr[GRCh37] **15q13.2q13.3(30737344_32514341) × 1**	15q13.2–15q13.3: 1.8 Mb deletion	*CHRNA7*	15q13.3 microdeletion syndrome	
506	4 year old male with global developmental delay, family history of intellectual disabilities	arr[GRCh37] **15q13.2q13.3(30936285_32514341) × 1**	15q13.2–15q13.3: 1.6 Mb deletion	*CHRNA7*	15q13.3 microdeletion syndrome	
453	6 year old male with global developmental delay, hypopigmentation	arr[GRCh37] 15q11.2(22750305_23272733) × 1, **15q13.3(32018731_32514341) × 1**, 10q26.12q26.2(122879869_128014502) × 2 hmz	15q11.2: 522 Kb deletion; 15q13.3: 496 Kb deletion; 10q26.12q26.2: 5.1 Mb LOH	*CHRNA7*	15q13.3 microdeletion syndrome	
313	8 year old female with mixed receptive-expressive language disorder, epilepsy, language regression	arr[GRCh37] **16p11.2(29595483_30198151) × 3**	16p11.2: 603 Kb duplication	*TBX6, KIF22, PRRT2*	16p11.2 microduplication	(D’Angelo et al., 2016; Kumar et al., 2009; Shinawi et al., 2010; Weiss et al., 2008)
488	14 year old male with congenital hypogonadotropic hypogonadism, micropenis, autism spectrum disorder	arr[GRCh37] **16p11.2(29595483_29733442) × 4,** **16p11.2(29763089_30198151) × 4**	16p11.2: 138 Kb triplication 16p11.2: 435 Kb triplication	*TBX6, KIF22, PRRT2*	16p11.2 triplication	This study
31	4 year old female with speech delay, low carnitine, language disorder	arr[GRCh37] **16p13.11(14968859_16291983) × 1**	16p13.11: 1.3 Mb deletion	*NDE1, MYH11, PXE, ABCC6*	16p13.1 deletion	This study; CNVs overlap with the 1.5 Mb neurocognitive susceptibility locus on 16p13.11 (Liu et al., 2012; Tan et al., 2017)
129	12 year old male with global developmental delay, obesity with serious comorbidity and body mass index (BMI) greater than 99th percentile for age in pediatric patient, Charcot-Marie-Tooth disease (CMT), overgrowth syndrome	arr[GRCh37] **17p12(14095309_15471179) × 3**	17p12: 1.4 Mb duplication	*PMP22*	Charcot-Marie-Tooth disease type 1A (CMT1A)	(Laura et al., 2019; Morena et al., 2019; Pareyson & Marchesi, 2009)
510	8 year old female with club foot, ptosis, speech disturbance, intermittent exotropia, balance problem, weakness, lack of coordination, mixed receptive-expressive language disorder, fine motor impairment, sensory processing difficulty, global developmental delay	arr[GRCh37] **18p11.32(13034_1324288) × 1,** **18q23(73690111_78015180) × 1**	18p11.32: 1.3 Mb deletion; 18q23: 4.3 Mb deletion	*CTDP1, TXNL4A, MBP*	Ring chromosome 18	This study; Monosomy of chr 18 and ring chr 18 with different breakpoints reported previously (Benini et al., 2012; Carter et al., 2015)
411	4 year old male with autism spectrum disorder, global developmental delay, mixed receptive-expressive language disorder	arr[GRCh37] **21p11.2q22.3(10827533_48100155) × 2–3**	21p11.2q22.3 Whole chromosome mosaic duplication	Whole chromosome with many genes	Mosaic trisomy 21	(Papavassiliou et al., 2015)
193	15 year old female with developmental delay, moderate persistent asthma, eczema, allergic rhinitis, spells, spell of altered consciousness	arr[GRCh37] **21q22.3(44210786_48100155) × 1**	21q22.3: 3.9 Mb deletion	*CSTB*	21q22.3 deletion	(Assenza et al., 2017; Ciocca et al., 2015; Poelmans et al., 2009)
277	3 year old male with global developmental delay, seizure, postaxial polydactyly in both hands, self-injurious behavior, autism	arr[GRCh37] 1p31.1(71987496_72370307) × 1, **22q11.21(18844632_21463730) × 1 mat**	1p31.1: 383 Kb deletion, 22q11.2: 2.6 Mb deletion	*TBX1, PRODH, COMT, SEPT5*	22q11.2 deletion syndrome (LCR22A-D)	(McDonald-McGinn et al., 2015; Zinkstok et al., 2019)
382	6 year old male with developmental delay, mixed receptive-expressive language disorder	arr[GRCh37] **22q11.21(18889490_21463730) × 1**	22q11.21: 2.6 Mb deletion	*TBX1, PRODH, COMT, SEPT5*	22q11.2 deletion syndrome (LCR22A-D)	
291	2 year old male with global developmental delay	arr[GRCh37] **22q11.21(18640300_21462353) × 3**	22q11.2: 2.8 Mb duplication	*TBX1*	22q11.2 duplication syndrome	(Kylat, 2018; Portnoi, 2009; Vyas et al., 2019)
147	2 year old male with global developmental delay, failure to thrive, hypotonia	arr[GRCh37] **Xp21.1(31737146_31759752) × 0**	Xp21.1: 23 Kb deletion	*DMD*	Duchenne muscular dystrophy	(Juan-Mateu et al., 2015; Muntoni et al., 2003; Takeshima et al., 2010)
416	6 year old male with accommodative esotropia, hypermetropia, speech disturbances	arr[GRCh37] **Xp21.1(31764087_31864634) × 0**	Xp21.1: 100.5 Kb deletion	*DMD*	Duchenne/Becker muscular dystrophy	
198	12 year old male with macrocephaly, epilepsy, autism	arr[GRCh37] **Xq28(152662222_153524347) × 2**	Xq28: 862 Kb duplication	*MECP2*	MECP2 duplication syndrome	(Lim et al., 2017; Ward et al., 2018)
211	4 year old male with autism spectrum disorder, mixed receptive-expressive language disorder, global developmental delay, sensory processing difficulty, disturbance in sleep behavior, disruptive behavior, insomnia	arr[GRCh37] **Xp22.31(6456940_8135053) × 0**	Xp22.31: 1.7 Mb deletion	*STS, VCX3A, XLI*	STS deficiency or X-linked ichthyosis (XLI)	(Ben Khelifa et al., 2013; Fernandes et al., 2010; Hand et al., 2015)
308	2 year old male with global developmental delay, hypotonia and seizures	arr[GRCh37] **Xp22.33q28(60814_155236712) × 2**	Xp22.33–Xq28: whole chromosome duplication	Whole chromosome with many genes	Klinefelter syndrome	(Crawford & Dearmun, 2017; Samango-Sprouse et al., 2020)
321	4 year old male with autism spectrum disorder, global developmental delay	arr[GRCh37] **Xp22.33q28(60814_155236712) × 2**	Xp22.33–Xq28: whole chromosome duplication	Whole chromosome with many genes	Klinefelter syndrome	

Patient identification number (ID), clinical information, International System for Human Cytogenetic Nomenclature (ISCN-2016) nomenclature, chromosome locations and size of alterations, genetic diagnosis and literature search results have been listed in the table. For patients with more than one CNV reported, the bolded CNV stands for the pathogenic/likely pathogenic CNV and the rest stand for Unknown Significance CNVs. See references in supplementary data

**Fig. 4:**
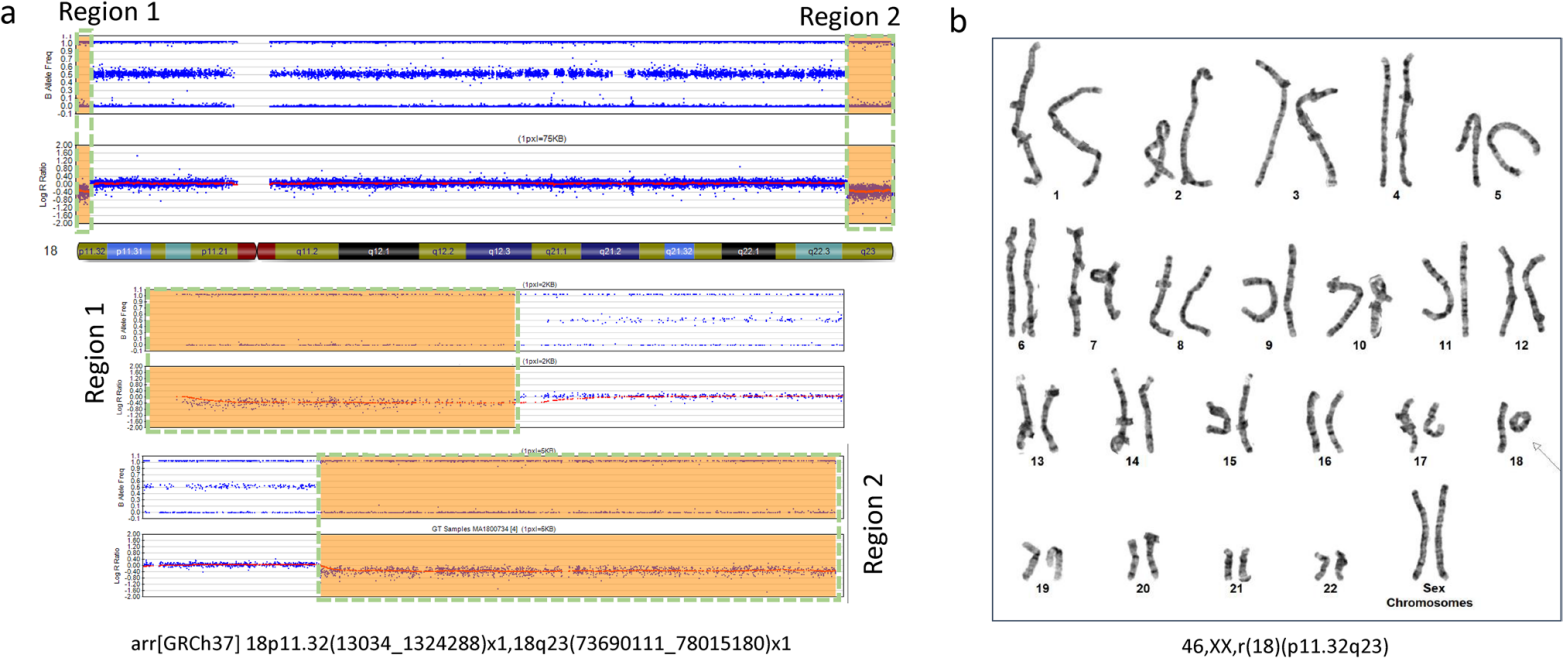
SNP microarray and karyotype analyses identified a ring chromosome 18 in a patient with club foot, ptosis, speech disturbance and global developmental delay. **a** Results from GenomeStudio shows two terminal deletions for chromosome 18, which suggested a ring chromosome 18. Region 1 corresponds to 18p11.32(13034_1324288) × 1 and region 2 corresponds to 18q23(73690111_78015180) × 1. **b** High resolution blood chromosome confirmed the ring chromosome 18 with a karyotype of 46,XX,r(18)(p11.32q23), which is indicated by the arrow

There are 30 pathogenic/likely pathogenic CNVs detected in 27 patients with a possible genetic diagnosis. Some patients carry more than one pathogenic/likely pathogenic CNVs. One patient (Pt 319, Table 1) with global developmental delay, speech delay, dysmorphic craniofacial features was identified with a 1.7 Mb duplication of 5p15.33 and a 2.2 Mb deletion of 18p11.32, which corresponded to a derivative chromosome 18 due to an unbalanced translocation between the short arms of chromosomes 5 and 18. Another patient was identified with two terminal deletions (1.3 Mb and 4.3 Mb) on both ends of chromosome 18. This patient presented with club foot, ptosis, speech disturbance, intermittent exotropia, balance problem, weakness, lack of coordination, mixed receptive-expressive language disorder, fine motor impairment, sensory processing difficulty and global developmental delay (Pt 510, Table 1; Fig. 4a). A ring 18 chromosome was suspected and was confirmed by the follow-up chromosome analysis on this patient with a karyotype of 46,XX,r(18)(p11.32q23) (Fig. 4b).

**Fig. 5:**
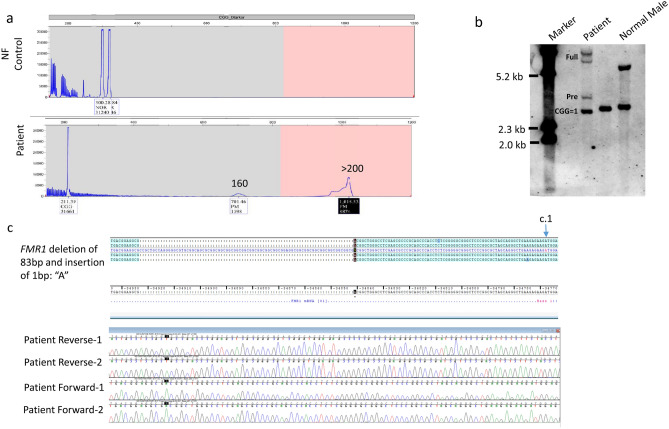
Mosaicism for a full mutation, premutation, and deletion of the CGG repeats in a male patient diagnosed with Fragile X syndrome. **a** Results of AmplideX *FMR1* PCR showed mosaic CGG amplification with 160 and > 200 repeats. In addition, there is a peak suggesting a deletion of CGG repeat (↑) in comparison of the normal female (NF) control. **b** Southern blot analysis detected differences in *FMR1* percent of methylation when comparing peripheral blood with control individuals (normal male and normal female). Full = full mutation, Pre = premutation. **c** Sanger sequencing results showed a 83 bp deletion and 1 bp insertion of A starting from the upstream of 5’ end of CGG repeat and resulting in only one CGG. The A of the start codon “ATG” has been designated as c.1

According to the NDR workflow, Fragile X tests were performed in 484 patients (94.72%). Eight patients showed abnormal *FMR1* CGG expansion size with repeats numbers including gray zone (45–54) (6 patients), premutation (55–200) (1 patient), and full mutation (> 200) (1 patient). This full mutation male patient clinically presented with mixed receptive-expressive language disorder, fine motor development delay and tracheoesophageal fistula. He was identified to have mosaic *FMR1* mutation alleles including a full mutation allele (> 200 CGG repeats), a premutation allele (160 repeats), and a third allele with a smaller than normal repeat size in the 5′ UTR of *FMR1* (Fig. 5a). The full mutation allele identified in this individual exhibited an abnormal methylation pattern of the *FMR1* gene and this was confirmed by Southern blot analysis (Fig. 5b). Sanger sequencing analysis of the third allele showed that there was a 83-bp deletion containing CGG repeats and an insertion of a single base “A” which resulted in only one CGG repeat in the 5′ UTR of the *FMR1* gene (Fig. 5c). By report, his maternal second cousin had Fragile X syndrome. There was no report of tremor or ataxia in grandfather, and there was no report of primary ovarian insufficiency in other female family members.

The *MECP2* test has been more utilized after we launched the NDR algorithm. In the past, when the *MECP2* test was just a stand-alone sequencing test, it was ordered, on average, on about 22 patients per year. After the launch of NDR algorithm, it has been ordered 3–4 times more than before as part of the NDR algorithm. A total of 101 patients (19.76%) were reflexed to *MECP2* sequencing analysis in our cohort. Two patients had heterozygous pathogenic missense variants c.467A > G (p.Asp156Gly) and c.473C > T (p.Thr158Met), respectively, which leads to a diagnosis of Rett syndrome. Two other patients were found to have a heterozygous variant of uncertain significance, c.824 T > C (p.Val275Ala) and c.-187_-186del (Table 2). Another patient detected with an abnormal *MECP2* result was a 12 year-old male patient with macrocephaly, epilepsy, and ASD. First-tier CMA analysis detected an 862 Kb duplication at Xq28, which included *MECP2*, and he was subsequently diagnosed with MECP2 Duplication Syndrome (Pt 198, Table 1). For the 99 patients tested for *PTEN* sequencing analysis, all of them returned with negative results.

**Table 2:** Summary of reportable *MECP2* sequence variants detected in 4 out of 101 patients with NDDs

Patient ID	Clinical presentation	Age of testing	Sex	Variant/Zygosity	Variant classification	gnomAD	ClinVar (Classification)	Publications/HGMD	AA/nt Conser-vation	SIFT/MutationTaster/Polyphen-2
358	Iron deficiency anemia, bilateral refractive amblyopia, bilateral Myopia, gross motor delay, hypotonia, global developmental delay	2 years old	Female	NM_004992.3: c.467A > G (p.Asp156Gly)/Het	PATH	Absent	Variant ID: 143583 (VUS)	Reported in patients with Rett Syndrome (Laccone et al., 2001; Trappe et al., 2001). Functional studies suggest D156G impairs transcription suppress activity (Kudo et al., 2003). Same codon different amino acid change listed in HGMD as disease causing variant HGMD: CM011798 (DM, Rett syndrome)	Highly conserved AA	Deleterious/Disease causing/Probably damaging
18	Autism spectrum disorder, global developmental delay	1 year old	Female	NM_004992.3: c.473C > T (p.Thr158Met)/Het	PATH	Absent	Variant ID: 11811 (PATH/LIKELY PATH)	Reported in multiple females with both classic and atypical Rett syndrome and some patients don’t meet clinical criteria for Rett syndrome (Neul et al., 2008; Percy et al., 2007). Reported in RettBASE: http://mecp2.chw.edu.au/mecp2/mecp2_home.php Functional studies suggest that T158M impairs normal protein function (Kucukkal et al., 2015; Kudo et al., 2003) HGMD: CM992178 (DM, Rett syndrome)	Highly conserved AA	Deleterious/Disease causing/Probably damaging
98	Speech disturbances, language impairment, developmental delay, autism spectrum disorder, sensory sensitivities	5 years old	Female	NM_004992.3: c.824 T > C (p.Val275Ala)/Het	VUS	0.0012% in European (non-Finnish); 0.0005% in global, 0 hemi	Variant ID: 431840 (VUS)	Reported in a patient with classic Rett syndrome (Petel-Galil et al., 2006) Not in HGMD	Moderately conserved AA	Tolerated/Disease causing/Benign
215	Autism spectrum disorder, mixed receptive-expressive language disorder, global developmental delay	5 years old	Female	NM_004992.3: c.-187_-186del/Het Same with NM_001110792.1: c.-27_-26del/Het	VUS	0.0579% in European (non-Finnish), 3 Hemi; 0.0356% in global	Not reported	Reported in a female patient with mental retardation, not observed in controls (Harvey et al., 2007) HGMD: CD075460 (DM?, Mental retardation)	Nucleotides conserved	N/A

Mutation nomenclature is based on the recommendation by Human Genome Variation Society (HGVS) that nucleotide + 1 is designated the A of the ATG-translation initiation codon. *AA* amino acid; *nt* nucleotide; *gnomAD* genome aggregation database; *HGMD* human gene mutation database, *PATH* pathogenic; *VUS* variant of unknown significance. See references in supplementary data

Through reviewing the results of our NDR reflective tests in the 511 patients with NDDs, we found that the diagnostic yield of NDR was 5.87% in our patient cohort, and the most often causes of NDDs were 15q13.3 microdeletion syndrome, 22q11.2 deletion syndrome, 16p11.2 duplication syndrome, as well as pathogenic variants, including CNVs, in *MECP2*.

## Discussion

Neurodevelopmental disorders are the most common medical conditions in pediatric population. Identifying the underlying etiology is important in the care of patients with NDDs including early intervention and clinical management, directing the patients and families to disease-specific supports and resources, informing prognosis, and recurrent risk assessment. Historically, the majority of NDD patients at our institution had concurrent orders placed for Fragile X testing, chromosome analysis, and SNP microarray analysis. The *MECP2* and *PTEN* sequencing tests were under-utilized. This NDR algorithm allows clinicians to place a single order that includes all of the testing recommended by ACMG for patients with NDDs, including testing for copy number changes, Fragile X syndrome, Rett syndrome, and *PTEN* related macrocephaly/autism syndrome (Schaefer et al., 2013). This algorithm was particularly helpful for the most common ordering providers who are from the Department of Developmental and Behavioral Pediatrics at CCHMC, who are not geneticists. The NDR was designed as a reflexive algorithm with the test with the highest diagnostic yield done first, then reflexing to downstream tests if prior testing is negative/normal. Starting with the highest yield test, the automatic reflexive nature of this algorithm warrants a saving in health care, comparing to the cost when these tests were ordered concurrently. Ordering these tests in a sequential order manually will have the same cost of saving effect. However, this automatic reflexive algorithm will save time in the overall diagnostic odyssey and is more user-friendly when compared with the manual sequential order of these tests. The NDR algorithm also promoted a greater utilization of the *MECP2* testing in NDD patients, especially for patients with atypical Rett syndrome, and further identified the genetic etiology for two patients in our cohort. These patients might be undiagnosed if the historical routine tests were ordered. Although the *PTEN* analysis in our cohort did not identify any positive cases, a negative result reduces the likelihood of *PTEN* as the genetic etiology for a patient with NDD, which is very meaningful for the patient care, since PTEN hamartoma tumor syndrome is associated with increased risk for certain cancers (breast, thyroid, renal cell, etc.).

One of the goals for this study was to review and evaluate the diagnostic yield of clinically ordered NDR tests. Our data show a 5.87% diagnostic yield, with the majority of positive cases (5.28%) diagnosed by CMA in the first-tier of testing. It has been reported that the diagnostic yield of CMA ranged from 4.5 to 28.0% in 19 studies of ID/DD and ranged from 1.5% to 20.5% in 11 studies with ASD (Savatt & Myers, 2021). Although our study did not separate ID/DD from ASD, the diagnostic yield of CMA in our cohort fell right into the previously reported diagnostic yield range of both. The most often causes of NDDs in our cohort from the first-tier CMA analysis were 15q13.3 microdeletion syndrome, 22q11.2 deletion syndrome, and 16p11.2 duplication syndrome. These findings were consistent with the “hot spots” autism loci reported in the 2013 ACMG practice guideline (Schaefer et al., 2013).

An interesting finding in our cohort that we would like to discuss is the ring chromosome. We detected by CMA and confirmed by high resolution chromosomal analysis one patient carrying a ring chromosome 18 with a karyotype 46,XX,r(18)(p11.32q23). Ring chromosome, which arises following breakage and rejoining in both chromosome arms, has been reported for all human chromosomes with an estimated frequency between 1/30,000 and 1/60,000 (Heydari et al., 2014). Carriers of ring chromosomes may have variable degrees of symptoms, from asymptomatic to serious defects in physical and intellectual development. Common features of patients with ring chromosome syndrome include short stature and developmental delay (Guilherme et al., 2013; Pristyazhnyuk & Menzorov, 2018). Although ring 18 chromosome with the exact breakpoints has not been reported so far, patients with partial monosomy 18p or ring 18 with different breakpoints have been reported with clinical features including difficulties in resisting infections, holoprosencephaly, micrognathia, tooth decay, ptosis, delayed development, intellectual disability, hypotonia, failure to thrive, short stature with growth hormone deficiencies, microcephaly, speech problems, hypertelorism, low-set ears, epicanthal folds, and cleft palate (Carter et al., 2015; Chen et al., 2010; Heydari et al., 2014; Stankiewicz et al., 2001; Timur et al., 2004). The signs and symptoms associated with a ring chromosome 18 depend on how much genetic material is lost from each arm of the chromosome. A critical gene is myelin basic protein (*MBP*; OMIM: 159430), which is located at the deleted region of 18q23 (Harauz et al., 2009). *MBP* gene encodes a protein which is incorporated in oligodendrocytes and Schwann cells myelin sheets. It has been reported as a candidate gene in a 2.5 year-old male, with an abnormal chromosome karyotype of 46,XY,r(18)(p11.32q21.32), who had overlapping features with our patient, including cleft lip, club foot and mild developmental delay (Heydari et al., 2014).

It was suggested that molecular single gene testing for Fragile X, Rett Syndrome, and *PTEN* will complement CMA and/or traditional cytogenetics in a clinical setting for neurodevelopmental/ASD disorder diagnosis (Schaefer et al., 2013). In this study, fragile X syndrome (FXS) testing identified mosaic *FMR1* full mutation alleles in a male patient. This patient carried a full mutation allele, a premutation allele, and an allele with one CGG repeat that resulted from an indel in the 5′ UTR region of the *FMR* gene. Deletions found in the mosaic state in full mutation males have been reported but are very rare and are typically larger than the deletion identified in this patient (Coffee et al., 2008; de Graaff et al., 1995; de Vries et al., 1993; Goncalves et al., 2016; Mannermaa et al., 1996). The phenotypic consequence of this indel in the mosaic state is currently unknown. Although we only found one patient with FXS, since ASD is present in 50–70% of individuals with FXS, a negative test result can exclude the diagnosis of FXS and allow subsequent reflex tests or other genetic tests to be performed.

*PTEN* is a well-known gene associated with ASD and macrocephaly (Butler et al., 2005; Varga et al., 2009; Zhou & Parada, 2012) and has been recommended by the ACMG practice guideline in identification of the etiology of ASD (Schaefer et al., 2013). Based on the ACMG practice guideline, we included *PTEN* sequencing in our NDR algorithm. When we initially established the NDR reflexing algorithm, *PTEN* analysis was set as the first-tier testing for patients who presented with macrocephaly, followed by Fragile X testing, and finally SNP microarray analysis. However, all the 40 patients with macrocephaly who received *PTEN* testing returned with negative results (data not shown). Therefore, we adjusted the algorithm as indicated in Fig. 1 to move *PTEN* sequencing to the third tier. Interestingly, in the current study, none of the 99 patients with macrocephaly were positive for pathogenic variants by *PTEN* sequence analysis. This “lower than expected” yield from *PTEN* sequencing may be due to the bias in defining macrocephaly or the fact that many macrocephalic patients in our cohort may have NDD other than ASD, which is the main symptom associated with *PTEN.* While this supports the decision to move *PTEN* to the end of the algorithm, given the extremely low yield of *PTEN* testing, we are considering replacing it with a more comprehensive next generation sequencing test which covers more genes and has a higher yield in NDDs. Single gene *PTEN* sequencing will still be available for clinicians to order if there is a strong clinical indication for a disorder associated with variants in this gene.

In summary, we show here that the NDR algorithm can effectively establish the genetic diagnosis for patients with NDDs, especially using CMA as a first-tier test following the ACMG guideline published in 2010 (Miller et al., 2010). With the advance of next-generation sequencing (NGS) technology and its implementation in clinical genetic laboratories, tests utilizing a combination of NGS and microarray have shown higher diagnostic yield in NDDs. In a recent study, a cohort of 8565 patients with epilepsy and NDDs tested by NGS and aCGH identified a genetic etiology in 15.4% of patients (Lindy et al., 2018). A meta-analysis comparing the yield of exome sequencing (ES) in NDDs with that of CMA showed that ES’s yield was markedly greater than CMA and a consensus was proposed to place ES as the first-tier clinical test in a diagnostic algorithm for unexplained NDDs (Srivastava et al., 2019). Recently, a new ACMG guideline was published recommending that exome and genome sequencing should be considered as a first- or second-tier test for patients with one or more congenital anomalies (CA)/DD/ID (Manickam et al., 2021).

Our laboratory recently launched an ASD/ID/DD exome slice panel with the option to reflex to WES, including trio analysis (proband and parents). We will monitor the performance of this exome slice panel with option reflex to WES test and plan to incorporate it into our NDR algorithm to continuously improve the diagnosis of NDDs. The field of clinical genetic testing is rapidly changing with advances in technology, such as genome sequencing, transcriptome sequencing, genome-wide methylation analysis, etc., which makes additional genetic tests available and affordable and more genes discovered in association with ASD/ID/DD. It is necessary for clinical genetic laboratories to continue to update genetic testing algorithms to increase the diagnosis of NDDs accordingly when new technologies become clinically available.

## Supplementary Information

Below is the link to the electronic supplementary material.

Supplementary file1 (DOCX 28 kb)
