# Supplementary material for: Genetic Testing in Patients with Neurodevelopmental Disorders: Experience of 511 Patients at Cincinnati Children's Hospital Medical Center
Source: J Autism Dev Disord. 2021 Nov 13;52(11):4828–42. doi: 10.1007/s10803-021-05337-6 (PMC9556427; doi:10.1007/s10803-021-05337-6)
Supplement: Supplementary file 1 — Supplementary file1 (DOCX 28 kb) [file 10803_2021_5337_MOESM1_ESM.docx]

**Supplemental Data**

**Methods**

**DNA extraction and SNP microarray analysis**

Genomic DNA was extracted either automatically using the Chemagic^™^ Magnetic Separation Module I instrument  (PerkinElmer, Waltham, MA) or manually using Qiagen Puregene kits (Qiagen, Germany). The microarray analysis was performed using the Infinium Assay with the Illumina CytoSNP-850Kv1.2 BeadChip platform (Illumina, San Diego, CA). This chip contains approximately 846,500 genome-wide markers, an overall average probe spacing of 1.8 kb and an average effective resolution of 18 kb to determine copy number change. Copy number changes ≥10 SNP markers are reviewed for clinical significance. B-allele frequency and log_2_R ratio were analyzed with Illumina Genome Studio V2009.2 software. Results are described using the International System of Human Cytogenetic Nomenclature (ISCN: 2016). Linear positions of abnormalities are listed according to the Human Genome Build (GRCh37: Feb. 2009(hg19)). Information regarding genes located within chromosomal regions are obtained from the Database of Genomic Variants (http://projects.tcag.ca/variation/), the human genome browser at UCSC (http://genome.ucsc.edu/), and the NCBI RefSeq Project (http://www.ncbi.nlm.nih.gov/RefSeq).

**Fragile X analysis**

Isolated genomic DNA was assayed for CGG-repeat expansion of the *FMR1* locus by analysis of DNA fragments generated by the AmplideX FMR1 polymerase chain reaction (PCR) (Asuragen, Austin, TX) and separated by capillary electrophoresis ABI 3500XL Genetic Analyzer (Applied Biosystems, Foster city, CA).  Normal and mutation categories of FMR1 allele were determined according to the ACMG guidelines with normal repeat size as 5-44, gray zone as 45-54, premutation as 55-200, and full mutation >200.  For Southern blot analysis, DNA underwent restriction digestion followed by gel electrophoresis, after which it was transferred to a membrane (Thermo Fisher Scientific, Waltham, MA). Ultra-violet (UV) light was used to permanently fix the DNA onto the membrane which was probe hybridized, and then using chemiluminescence, it was visualized on an imager (Roche, Indianapolis, IN).

***PTEN* and *MECP2* PCR and sequencing**

The entire coding region and exon/intron boundaries of genes were analyzed by PCR and bidirectional sequencing for *PTEN* (NM_000314.4) and *MECP2* (NM_004992.2) according to manufactory instructions (Roche, Indianapolis, IN). The sensitivity of DNA sequencing is over 99% for the detection of nucleotide base changes, small deletions and insertions in the regions analyzed. Mutations in regulatory regions or other untranslated regions are not detected by this test. Large deletions involving entire single exon or multiple exons, large insertions and genetic recombinational events may not be identified using these methods. Detailed methodology is available upon request.

**Blood chromosomes**

Chromosomal analysis was performed according to standard procedures using GTG-banding. Peripheral blood lymphocytes were cultured in RPMI 1640 medium enriched with FBS, phytohemagglutinin and L- glutamine (Thermo Fisher Scientific, Waltham, MA). The cells were cultured for 72 hours at 37°C. Cells were harvested by adding colcemid for 2 hours. Cells were exposed to a hypotonic solution (KCl 0.075 mol/L) and fixed with methanol/acetic acid (3:1) (vol/vol). Metaphase chromosome spreads were prepared and G-banding with the use of trypsin-Giemsa at a resolution of 550 bands was established. A minimum of 20 metaphases were examined from each patient. Chromosome abnormalities were described according to the ISCN 2016.

**References for Table 1:**

Amarillo, I. E., Li, W. L., Li, X., Vilain, E., & Kantarci, S. (2014). De novo single exon deletion of AUTS2 in a patient with speech and language disorder: a review of disrupted AUTS2 and further evidence for its role in neurodevelopmental disorders. *Am J Med Genet A*, *164A*(4), 958-965. <https://doi.org/10.1002/ajmg.a.36393>

Assenza, G., Benvenga, A., Gennaro, E., Tombini, M., Campana, C., Assenza, F., Di Pino, G., & Di Lazzaro, V. (2017). A novel c132-134del mutation in Unverricht-Lundborg disease and the review of literature of heterozygous compound patients. *Epilepsia*, *58*(2), e31-e35. <https://doi.org/10.1111/epi.13626>

Bayat, A., Kirchhoff, M., Madsen, C. G., & Kreiborg, S. (2018). Neonatal hyperinsulinemic hypoglycemia in a patient with 9p deletion syndrome. *Eur J Med Genet*, *61*(8), 473-477. <https://doi.org/10.1016/j.ejmg.2018.03.009>

Ben-Shachar, S., Lanpher, B., German, J. R., Qasaymeh, M., Potocki, L., Nagamani, S. C., Franco, L. M., Malphrus, A., Bottenfield, G. W., Spence, J. E., Amato, S., Rousseau, J. A., Moghaddam, B., Skinner, C., Skinner, S. A., Bernes, S., Armstrong, N., Shinawi, M., Stankiewicz, P., Patel, A., Cheung, S. W., Lupski, J. R., Beaudet, A. L., & Sahoo, T. (2009). Microdeletion 15q13.3: a locus with incomplete penetrance for autism, mental retardation, and psychiatric disorders. *J Med Genet*, *46*(6), 382-388. <https://doi.org/10.1136/jmg.2008.064378>

Ben Khelifa, H., Soyah, N., Ben-Abdallah-Bouhjar, I., Gritly, R., Sanlaville, D., Elghezal, H., Saad, A., & Mougou-Zerelli, S. (2013). Xp22.3 interstitial deletion: a recognizable chromosomal abnormality encompassing VCX3A and STS genes in a patient with X-linked ichthyosis and mental retardation. *Gene*, *527*(2), 578-583. <https://doi.org/10.1016/j.gene.2013.06.018>

Benini, R., Saint-Martin, C., Shevell, M. I., & Bernard, G. (2012). Abnormal myelination in ring chromosome 18 syndrome. *J Child Neurol*, *27*(8), 1042-1047. <https://doi.org/10.1177/0883073811430268>

Beunders, G., van de Kamp, J., Vasudevan, P., Morton, J., Smets, K., Kleefstra, T., de Munnik, S. A., Schuurs-Hoeijmakers, J., Ceulemans, B., Zollino, M., Hoffjan, S., Wieczorek, S., So, J., Mercer, L., Walker, T., Velsher, L., study, D. D. D., Parker, M. J., Magee, A. C., Elffers, B., Kooy, R. F., Yntema, H. G., Meijers-Heijboer, E. J., & Sistermans, E. A. (2016). A detailed clinical analysis of 13 patients with AUTS2 syndrome further delineates the phenotypic spectrum and underscores the behavioural phenotype. *J Med Genet*, *53*(8), 523-532. <https://doi.org/10.1136/jmedgenet-2015-103601>

Beunders, G., Voorhoeve, E., Golzio, C., Pardo, L. M., Rosenfeld, J. A., Talkowski, M. E., Simonic, I., Lionel, A. C., Vergult, S., Pyatt, R. E., van de Kamp, J., Nieuwint, A., Weiss, M. M., Rizzu, P., Verwer, L. E., van Spaendonk, R. M., Shen, Y., Wu, B. L., Yu, T., Yu, Y., Chiang, C., Gusella, J. F., Lindgren, A. M., Morton, C. C., van Binsbergen, E., Bulk, S., van Rossem, E., Vanakker, O., Armstrong, R., Park, S. M., Greenhalgh, L., Maye, U., Neill, N. J., Abbott, K. M., Sell, S., Ladda, R., Farber, D. M., Bader, P. I., Cushing, T., Drautz, J. M., Konczal, L., Nash, P., de Los Reyes, E., Carter, M. T., Hopkins, E., Marshall, C. R., Osborne, L. R., Gripp, K. W., Thrush, D. L., Hashimoto, S., Gastier-Foster, J. M., Astbury, C., Ylstra, B., Meijers-Heijboer, H., Posthuma, D., Menten, B., Mortier, G., Scherer, S. W., Eichler, E. E., Girirajan, S., Katsanis, N., Groffen, A. J., & Sistermans, E. A. (2013). Exonic deletions in AUTS2 cause a syndromic form of intellectual disability and suggest a critical role for the C terminus. *Am J Hum Genet*, *92*(2), 210-220. <https://doi.org/10.1016/j.ajhg.2012.12.011>

Bonaglia, M. C., Ciccone, R., Gimelli, G., Gimelli, S., Marelli, S., Verheij, J., Giorda, R., Grasso, R., Borgatti, R., Pagone, F., Rodriguez, L., Martinez-Frias, M. L., van Ravenswaaij, C., & Zuffardi, O. (2008). Detailed phenotype-genotype study in five patients with chromosome 6q16 deletion: narrowing the critical region for Prader-Willi-like phenotype. *Eur J Hum Genet*, *16*(12), 1443-1449. <https://doi.org/10.1038/ejhg.2008.119>

Carter, E., Heard, P., Hasi, M., Soileau, B., Sebold, C., Hale, D. E., & Cody, J. D. (2015). Ring 18 molecular assessment and clinical consequences. *Am J Med Genet A*, *167A*(1), 54-63. <https://doi.org/10.1002/ajmg.a.36822>

Ciocca, L., Digilio, M. C., Lombardo, A., D'Elia, G., Baban, A., Capolino, R., Petrocchi, S., Russo, S., Sirleto, P., Roberti, M. C., Marino, B., Angioni, A., & Dallapiccola, B. (2015). Hypoplastic left heart syndrome and 21q22.3 deletion. *Am J Med Genet A*, *167A*(3), 579-586. <https://doi.org/10.1002/ajmg.a.36914>

Crawford, D., & Dearmun, A. (2017). Klinefelter syndrome. *Nurs Child Young People*, *29*(6), 19. <https://doi.org/10.7748/ncyp.29.6.19.s21>

D'Angelo, D., Lebon, S., Chen, Q., Martin-Brevet, S., Snyder, L. G., Hippolyte, L., Hanson, E., Maillard, A. M., Faucett, W. A., Mace, A., Pain, A., Bernier, R., Chawner, S. J., David, A., Andrieux, J., Aylward, E., Baujat, G., Caldeira, I., Conus, P., Ferrari, C., Forzano, F., Gerard, M., Goin-Kochel, R. P., Grant, E., Hunter, J. V., Isidor, B., Jacquette, A., Jonch, A. E., Keren, B., Lacombe, D., Le Caignec, C., Martin, C. L., Mannik, K., Metspalu, A., Mignot, C., Mukherjee, P., Owen, M. J., Passeggeri, M., Rooryck-Thambo, C., Rosenfeld, J. A., Spence, S. J., Steinman, K. J., Tjernagel, J., Van Haelst, M., Shen, Y., Draganski, B., Sherr, E. H., Ledbetter, D. H., van den Bree, M. B., Beckmann, J. S., Spiro, J. E., Reymond, A., Jacquemont, S., Chung, W. K., Cardiff University Experiences of Children With Copy Number Variants, S., p11.2 European, C., & Simons Variation in Individuals Project, C. (2016). Defining the Effect of the 16p11.2 Duplication on Cognition, Behavior, and Medical Comorbidities. *JAMA Psychiatry*, *73*(1), 20-30. <https://doi.org/10.1001/jamapsychiatry.2015.2123>

D'Arienzo, A., Andreani, L., Sacchetti, F., Colangeli, S., & Capanna, R. (2019). Hereditary Multiple Exostoses: Current Insights. *Orthop Res Rev*, *11*, 199-211. <https://doi.org/10.2147/ORR.S183979>

Fernandes, N. F., Janniger, C. K., & Schwartz, R. A. (2010). X-linked ichthyosis: an oculocutaneous genodermatosis. *J Am Acad Dermatol*, *62*(3), 480-485. <https://doi.org/10.1016/j.jaad.2009.04.028>

Hand, J. L., Runke, C. K., & Hodge, J. C. (2015). The phenotype spectrum of X-linked ichthyosis identified by chromosomal microarray. *J Am Acad Dermatol*, *72*(4), 617-627. <https://doi.org/10.1016/j.jaad.2014.12.020>

Hoppman-Chaney, N., Wain, K., Seger, P. R., Superneau, D. W., & Hodge, J. C. (2013). Identification of single gene deletions at 15q13.3: further evidence that CHRNA7 causes the 15q13.3 microdeletion syndrome phenotype. *Clin Genet*, *83*(4), 345-351. <https://doi.org/10.1111/j.1399-0004.2012.01925.x>

Jennes, I., Entius, M. M., Van Hul, E., Parra, A., Sangiorgi, L., & Wuyts, W. (2008). Mutation screening of EXT1 and EXT2 by denaturing high-performance liquid chromatography, direct sequencing analysis, fluorescence in situ hybridization, and a new multiplex ligation-dependent probe amplification probe set in patients with multiple osteochondromas. *J Mol Diagn*, *10*(1), 85-92. <https://doi.org/10.2353/jmoldx.2008.070086>

Juan-Mateu, J., Gonzalez-Quereda, L., Rodriguez, M. J., Baena, M., Verdura, E., Nascimento, A., Ortez, C., Baiget, M., & Gallano, P. (2015). DMD Mutations in 576 Dystrophinopathy Families: A Step Forward in Genotype-Phenotype Correlations. *PLoS One*, *10*(8), e0135189. <https://doi.org/10.1371/journal.pone.0135189>

Kasher, P. R., Schertz, K. E., Thomas, M., Jackson, A., Annunziata, S., Ballesta-Martinez, M. J., Campeau, P. M., Clayton, P. E., Eaton, J. L., Granata, T., Guillen-Navarro, E., Hernando, C., Laverriere, C. E., Lieden, A., Villa-Marcos, O., McEntagart, M., Nordgren, A., Pantaleoni, C., Pebrel-Richard, C., Sarret, C., Sciacca, F. L., Wright, R., Kerr, B., Glasgow, E., & Banka, S. (2016). Small 6q16.1 Deletions Encompassing POU3F2 Cause Susceptibility to Obesity and Variable Developmental Delay with Intellectual Disability. *Am J Hum Genet*, *98*(2), 363-372. <https://doi.org/10.1016/j.ajhg.2015.12.014>

Kumar, R. A., Marshall, C. R., Badner, J. A., Babatz, T. D., Mukamel, Z., Aldinger, K. A., Sudi, J., Brune, C. W., Goh, G., Karamohamed, S., Sutcliffe, J. S., Cook, E. H., Geschwind, D. H., Dobyns, W. B., Scherer, S. W., & Christian, S. L. (2009). Association and mutation analyses of 16p11.2 autism candidate genes. *PLoS One*, *4*(2), e4582. <https://doi.org/10.1371/journal.pone.0004582>

Kylat, R. I. (2018). 22q11.2 Microduplication: An Enigmatic Genetic Disorder. *J Pediatr Genet*, *7*(3), 138-142. <https://doi.org/10.1055/s-0038-1655754>

Laura, M., Pipis, M., Rossor, A. M., & Reilly, M. M. (2019). Charcot-Marie-Tooth disease and related disorders: an evolving landscape. *Curr Opin Neurol*, *32*(5), 641-650. <https://doi.org/10.1097/WCO.0000000000000735>

Lim, Z., Downs, J., Wong, K., Ellaway, C., & Leonard, H. (2017). Expanding the clinical picture of the MECP2 Duplication syndrome. *Clin Genet*, *91*(4), 557-563. <https://doi.org/10.1111/cge.12814>

Liu, J. Y., Kasperaviciute, D., Martinian, L., Thom, M., & Sisodiya, S. M. (2012). Neuropathology of 16p13.11 deletion in epilepsy. *PLoS One*, *7*(4), e34813. <https://doi.org/10.1371/journal.pone.0034813>

Liu, Y., Zhao, D., Dong, R., Yang, X., Zhang, Y., Tammimies, K., Uddin, M., Scherer, S. W., & Gai, Z. (2015). De novo exon 1 deletion of AUTS2 gene in a patient with autism spectrum disorder and developmental delay: a case report and a brief literature review. *Am J Med Genet A*, *167*(6), 1381-1385. <https://doi.org/10.1002/ajmg.a.37050>

Lowther, C., Costain, G., Stavropoulos, D. J., Melvin, R., Silversides, C. K., Andrade, D. M., So, J., Faghfoury, H., Lionel, A. C., Marshall, C. R., Scherer, S. W., & Bassett, A. S. (2015). Delineating the 15q13.3 microdeletion phenotype: a case series and comprehensive review of the literature. *Genet Med*, *17*(2), 149-157. <https://doi.org/10.1038/gim.2014.83>

McDonald-McGinn, D. M., Sullivan, K. E., Marino, B., Philip, N., Swillen, A., Vorstman, J. A., Zackai, E. H., Emanuel, B. S., Vermeesch, J. R., Morrow, B. E., Scambler, P. J., & Bassett, A. S. (2015). 22q11.2 deletion syndrome. *Nat Rev Dis Primers*, *1*, 15071. <https://doi.org/10.1038/nrdp.2015.71>

Morena, J., Gupta, A., & Hoyle, J. C. (2019). Charcot-Marie-Tooth: From Molecules to Therapy. *Int J Mol Sci*, *20*(14). <https://doi.org/10.3390/ijms20143419>

Muntoni, F., Torelli, S., & Ferlini, A. (2003). Dystrophin and mutations: one gene, several proteins, multiple phenotypes. *Lancet Neurol*, *2*(12), 731-740. <https://doi.org/10.1016/s1474-4422(03)00585-4>

Papavassiliou, P., Charalsawadi, C., Rafferty, K., & Jackson-Cook, C. (2015). Mosaicism for trisomy 21: a review. *Am J Med Genet A*, *167A*(1), 26-39. <https://doi.org/10.1002/ajmg.a.36861>

Pareyson, D., & Marchesi, C. (2009). Diagnosis, natural history, and management of Charcot-Marie-Tooth disease. *Lancet Neurol*, *8*(7), 654-667. <https://doi.org/10.1016/S1474-4422(09)70110-3>

Pober, B. R. (2010). Williams-Beuren syndrome. *N Engl J Med*, *362*(3), 239-252. <https://doi.org/10.1056/NEJMra0903074>

Poelmans, G., Engelen, J. J., Van Lent-Albrechts, J., Smeets, H. J., Schoenmakers, E., Franke, B., Buitelaar, J. K., Wuisman-Frerker, M., Erens, W., Steyaert, J., & Schrander-Stumpel, C. (2009). Identification of novel dyslexia candidate genes through the analysis of a chromosomal deletion. *Am J Med Genet B Neuropsychiatr Genet*, *150B*(1), 140-147. <https://doi.org/10.1002/ajmg.b.30787>

Portnoi, M. F. (2009). Microduplication 22q11.2: a new chromosomal syndrome. *Eur J Med Genet*, *52*(2-3), 88-93. <https://doi.org/10.1016/j.ejmg.2009.02.008>

Samango-Sprouse, C. A., Yu, C., Porter, G. F., Tipton, E. S., Lasutschinkow, P. C., & Gropman, A. L. (2020). A review of the intriguing interaction between testosterone and neurocognitive development in males with 47,XXY. *Curr Opin Obstet Gynecol*, *32*(2), 140-146. <https://doi.org/10.1097/GCO.0000000000000612>

Samanta, D. (2017). Infantile spasms in Williams-Beuren syndrome with typical deletions of the 7q11.23 critical region and a review of the literature. *Acta Neurol Belg*, *117*(1), 359-362. <https://doi.org/10.1007/s13760-016-0635-0>

Shinawi, M., Liu, P., Kang, S. H., Shen, J., Belmont, J. W., Scott, D. A., Probst, F. J., Craigen, W. J., Graham, B. H., Pursley, A., Clark, G., Lee, J., Proud, M., Stocco, A., Rodriguez, D. L., Kozel, B. A., Sparagana, S., Roeder, E. R., McGrew, S. G., Kurczynski, T. W., Allison, L. J., Amato, S., Savage, S., Patel, A., Stankiewicz, P., Beaudet, A. L., Cheung, S. W., & Lupski, J. R. (2010). Recurrent reciprocal 16p11.2 rearrangements associated with global developmental delay, behavioural problems, dysmorphism, epilepsy, and abnormal head size. *J Med Genet*, *47*(5), 332-341. <https://doi.org/10.1136/jmg.2009.073015>

Sivasankaran, A., Kanakavalli, M. K., Anuradha, D., Samuel, C. R., & Kandukuri, L. R. (2016). Ring Chromosome 9 and Chromosome 9p Deletion Syndrome in a Patient Associated with Developmental Delay: A Case Report and Review of the Literature. *Cytogenet Genome Res*, *148*(2-3), 165-173. <https://doi.org/10.1159/000445862>

Spazzapan, P., Arnaud, E., Baujat, G., Nizon, M., Malan, V., Brunelle, F., & Di Rocco, F. (2016). Clinical and neuroradiological features of the 9p deletion syndrome. *Childs Nerv Syst*, *32*(2), 327-335. <https://doi.org/10.1007/s00381-015-2957-2>

Strunk, D., Weber, P., Rothlisberger, B., & Filges, I. (2016). Autism and intellectual disability in a patient with two microdeletions in 6q16: a contiguous gene deletion syndrome? *Mol Cytogenet*, *9*, 88. <https://doi.org/10.1186/s13039-016-0299-8>

Takeshima, Y., Yagi, M., Okizuka, Y., Awano, H., Zhang, Z., Yamauchi, Y., Nishio, H., & Matsuo, M. (2010). Mutation spectrum of the dystrophin gene in 442 Duchenne/Becker muscular dystrophy cases from one Japanese referral center. *J Hum Genet*, *55*(6), 379-388. <https://doi.org/10.1038/jhg.2010.49>

Tan, L., Bi, B., Zhao, P., Cai, X., Wan, C., Shao, J., & He, X. (2017). Severe congenital microcephaly with 16p13.11 microdeletion combined with NDE1 mutation, a case report and literature review. *BMC Med Genet*, *18*(1), 141. <https://doi.org/10.1186/s12881-017-0501-9>

Tassano, E., Accogli, A., Pavanello, M., Bruno, C., Capra, V., Gimelli, G., & Cuoco, C. (2016). Interstitial 9p24.3 deletion involving only DOCK8 and KANK1 genes in two patients with non-overlapping phenotypic traits. *Eur J Med Genet*, *59*(1), 20-25. <https://doi.org/10.1016/j.ejmg.2015.11.011>

Vyas, S., Constantino, J. N., & Baldridge, D. (2019). 22q11.2 duplication: a review of neuropsychiatric correlates and a newly observed case of prototypic sociopathy. *Cold Spring Harb Mol Case Stud*, *5*(6). <https://doi.org/10.1101/mcs.a004291>

Ward, D. I., Buckley, B. A., Leon, E., Diaz, J., Galegos, M. F., Hofherr, S., & Lewanda, A. F. (2018). Intellectual disability and epilepsy due to the K/L-mediated Xq28 duplication: Further evidence of a distinct, dosage-dependent phenotype. *Am J Med Genet A*, *176*(3), 551-559. <https://doi.org/10.1002/ajmg.a.38524>

Weiss, L. A., Shen, Y., Korn, J. M., Arking, D. E., Miller, D. T., Fossdal, R., Saemundsen, E., Stefansson, H., Ferreira, M. A., Green, T., Platt, O. S., Ruderfer, D. M., Walsh, C. A., Altshuler, D., Chakravarti, A., Tanzi, R. E., Stefansson, K., Santangelo, S. L., Gusella, J. F., Sklar, P., Wu, B. L., Daly, M. J., & Autism, C. (2008). Association between microdeletion and microduplication at 16p11.2 and autism. *N Engl J Med*, *358*(7), 667-675. <https://doi.org/10.1056/NEJMoa075974>

Ziats, M. N., Goin-Kochel, R. P., Berry, L. N., Ali, M., Ge, J., Guffey, D., Rosenfeld, J. A., Bader, P., Gambello, M. J., Wolf, V., Penney, L. S., Miller, R., Lebel, R. R., Kane, J., Bachman, K., Troxell, R., Clark, G., Minard, C. G., Stankiewicz, P., Beaudet, A., & Schaaf, C. P. (2016). The complex behavioral phenotype of 15q13.3 microdeletion syndrome. *Genet Med*, *18*(11), 1111-1118. <https://doi.org/10.1038/gim.2016.9>

Zinkstok, J. R., Boot, E., Bassett, A. S., Hiroi, N., Butcher, N. J., Vingerhoets, C., Vorstman, J. A. S., & van Amelsvoort, T. (2019). Neurobiological perspective of 22q11.2 deletion syndrome. *Lancet Psychiatry*, *6*(11), 951-960. <https://doi.org/10.1016/S2215-0366(19)30076-8>

**References for Table 2:**

Harvey, C. G., Menon, S. D., Stachowiak, B., Noor, A., Proctor, A., Mensah, A. K., Mnatzakanian, G. N., Alfred, S. E., Guo, R., Scherer, S. W., Kennedy, J. L., Roberts, W., Srivastava, A. K., Minassian, B. A., & Vincent, J. B. (2007). Sequence variants within exon 1 of MECP2 occur in females with mental retardation. *Am J Med Genet B Neuropsychiatr Genet*, *144B*(3), 355-360. <https://doi.org/10.1002/ajmg.b.30425>

Kucukkal, T. G., Yang, Y., Uvarov, O., Cao, W., & Alexov, E. (2015). Impact of Rett Syndrome Mutations on MeCP2 MBD Stability. *Biochemistry*, *54*(41), 6357-6368. <https://doi.org/10.1021/acs.biochem.5b00790>

Kudo, S., Nomura, Y., Segawa, M., Fujita, N., Nakao, M., Schanen, C., & Tamura, M. (2003). Heterogeneity in residual function of MeCP2 carrying missense mutations in the methyl CpG binding domain. *J Med Genet*, *40*(7), 487-493. <https://doi.org/10.1136/jmg.40.7.487>

Laccone, F., Huppke, P., Hanefeld, F., & Meins, M. (2001). Mutation spectrum in patients with Rett syndrome in the German population: Evidence of hot spot regions. *Hum Mutat*, *17*(3), 183-190. <https://doi.org/10.1002/humu.3>

Neul, J. L., Fang, P., Barrish, J., Lane, J., Caeg, E. B., Smith, E. O., Zoghbi, H., Percy, A., & Glaze, D. G. (2008). Specific mutations in methyl-CpG-binding protein 2 confer different severity in Rett syndrome. *Neurology*, *70*(16), 1313-1321. <https://doi.org/10.1212/01.wnl.0000291011.54508.aa>

Percy, A. K., Lane, J. B., Childers, J., Skinner, S., Annese, F., Barrish, J., Caeg, E., Glaze, D. G., & MacLeod, P. (2007). Rett syndrome: North American database. *J Child Neurol*, *22*(12), 1338-1341. <https://doi.org/10.1177/0883073807308715>

Petel-Galil, Y., Benteer, B., Galil, Y. P., Zeev, B. B., Greenbaum, I., Vecsler, M., Goldman, B., Lohi, H., Minassian, B. A., & Gak, E. (2006). Comprehensive diagnosis of Rett's syndrome relying on genetic, epigenetic and expression evidence of deficiency of the methyl-CpG-binding protein 2 gene: study of a cohort of Israeli patients. *J Med Genet*, *43*(12), e56. <https://doi.org/10.1136/jmg.2006.041285>

Trappe, R., Laccone, F., Cobilanschi, J., Meins, M., Huppke, P., Hanefeld, F., & Engel, W. (2001). MECP2 mutations in sporadic cases of Rett syndrome are almost exclusively of paternal origin. *Am J Hum Genet*, *68*(5), 1093-1101. <https://doi.org/10.1086/320109>
